# UK circulating strains of human parainfluenza 3: an amplicon based next generation sequencing method and phylogenetic analysis

**DOI:** 10.12688/wellcomeopenres.14730.2

**Published:** 2018-11-26

**Authors:** Anna Smielewska, Edward Emmott, Kyriaki Ranellou, Ashley Popay, Ian Goodfellow, Hamid Jalal

**Affiliations:** 1 Department of Pathology, University of Cambridge Addenbrooke's Hospital Cambridge, Cambridge, Cambridgeshire, CB20QQ, UK; 2 Cambridge University Hospitals NHS Foundation Trust Laboratory, Public Health England, Cambridge, Cambridgeshire, CB20QQ, UK; 3 Department of Bioengineering, Northeastern University, Boston, MA, 02115-5000, USA; 4 Eastern Field Epidemiology Unit, Institute of Public Health, Public Health England, Cambridge, Cambridgeshire, CB20SR, UK

**Keywords:** human parainfluenza 3, phylogenetics, epidemiology, circulating strains

## Abstract

**Background: **Human parainfluenza viruses type 3 (HPIV3) are a prominent cause of respiratory infection with a significant impact in both pediatric and transplant patient cohorts.  Currently there is a paucity of whole genome sequence data that would allow for detailed epidemiological and phylogenetic analysis of circulating strains in the UK. Although it is known that HPIV3 peaks annually in the UK, to date there are no whole genome sequences of HPIV3 UK strains available.

**Methods: **Clinical strains were obtained from HPIV3 positive respiratory patient samples collected between 2011 and 2015.  These were then amplified using an amplicon based method, sequenced on the Illumina platform and assembled using a new robust bioinformatics pipeline. Phylogenetic analysis was carried out in the context of other epidemiological studies and whole genome sequence data currently available with stringent exclusion of significantly culture-adapted strains of HPIV3.

**Results: **In the current paper we have presented twenty full genome sequences of UK circulating strains of HPIV3 and a detailed phylogenetic analysis thereof.  We have analysed the variability along the HPIV3 genome and identified a short hypervariable region in the non-coding segment between the M (matrix) and F (fusion) genes. The epidemiological classifications obtained by using this region and whole genome data were then compared and found to be identical.

**Conclusions: **The majority of HPIV3 strains were observed at different geographical locations and with a wide temporal spread, reflecting the global distribution of HPIV3. Consistent with previous data, a particular subcluster or strain was not identified as specific to the UK, suggesting that a number of genetically diverse strains circulate at any one time. A small hypervariable region in the HPIV3 genome was identified and it was shown that, in the absence of full genome data, this region could be used for epidemiological surveillance of HPIV3.

## Introduction

Human parainfluenza viruses (HPIV) are members of the family Paramyxoviridae and are subdivided into four types, which fall into two genera *Rubulavirus (types 2 and 4 and Respirovirus (types 1 and 3).* Human parainfluenza 3 (HPIV3) (recently renamed human respirovirus 3) is a negative strand non-segmented RNA virus of 15462 nucleotides in length. It consists of a core containing the RNA bound to the nucleocapsid protein (NP), the phosphoprotein (P) and the large RNA polymerase (L) surrounded by an envelope composed of the matrix protein (M) and a lipid bilayer. The haemagluttinin neuraminidase (HN) protein and the fusion (F) protein are found on the envelope surface and facilitate the binding of HPIV3 to the sialic acid receptors of the target cell via the haemagluttinin component of HN, the fusion (F) with the cell and the release of new viral particles via the neuraminidase component of HN ^1, 2^.

All four types of parainfluenza are significant causes of both upper and lower respiratory tract infections. Human parainfluenza 3 (HPIV3) has been identified as the most prevalent circulating parainfluenza serotype in the UK ^3^. It is an important respiratory pathogen with a broad spectrum of presentations and a significant impact both in the paediatric and the immunocompromised cohorts. In the former it is responsible for up to 6.8% of all paediatric admissions for respiratory presentations ^4^. In the immunocompromised population, the reported incidence of infection has varied between 5 and 12% with lower respiratory tract infections (LRTIs) and mortality of up to 75% being reported ^5, 6^. Transmission is by respiratory droplets and HPIV3 can persist up to 10 hours on non-absorbant surfaces ^7^. Prolonged shedding in vulnerable patient groups leads to outbreaks and an increased burden on the health services ^8, 9^. Although a number of previous studies have looked at circulating HPIV3 strains throughout the world, there is currently no genetic data on the circulating HPIV3 strains in the UK.

Previous phylogenetic analysis of HPIV3 has been based on components of the genome rather than full-length genome data. To date there appears to be no unified phylogenetic classification of HPIV3. Recently the HN gene has been used to characterize emerging strains as well as tracing outbreaks ^10– 14^. Automatic barcode gap discovery (ABGD) ^15^ was used to separate HPIV3 into 3 clusters with currently circulating strains confined to one of these. In another study it was shown that the F gene is equally valid for HPIV3 phylogenetic classification ^11^. The region directly preceding and overlapping with the start of the F genome has previously been identified as highly variable and has been historically used to trace outbreaks ^8, 9^.

Additionally there is currently little experimental evidence linking HPIV3 HN and F phenotype with variations in the adaptive immune response ^16, 17^ and currently no consensus on the connection between genotype and clinical pathogenesis of HPIV3. Therefore there is a clear need to rationalize the approach to the phylogenetic and epidemiological analysis of HPIV3.

To this end, in this study we have presented the genetic analysis of full genome sequences of twenty circulatingUK strains between the years 2011–2015. We have used this data, together with other full genome sequences available in the genebank to conduct a full genome phylogentic analysis of HPIV3. Although rapid metagenomic sequencing has been conducted in a small outbreak ^18^, given the relative expense of obtaining full genome data in a clinical setting we have identified a short hypervariable region in the HPIV3 genome and evaluated the reliability of using this segment for future phylogenetic analysis and potential epidemiological investigation.

## Methods

### Clinical samples

Clinical strains were obtained from HPIV3 positive respiratory patient samples collected between 2011 and 2015 by Public Health England (PHE) laboratory in a major teaching hospital. All identifiable information was removed prior to the study. Anonymous patient demographics such as age, sex, location, as well as date and type of the sample were retained where possible (ethics approval number 12/EE/0069).

All clinical strains were grown on PLC/PRF/5 human Alexander hepatoma cell line as described in a separate study ^19^ and underwent an additional passage for RNA harvesting. 43 samples were successfully grown and 20 clinical strains were selected for subsequent sequence analysis. Laboratory strain MK9 obtained from PHE cultures was used as a reference strain for sequencing pipeline validation.

### RNA extraction and amplification

**Figure 1.**
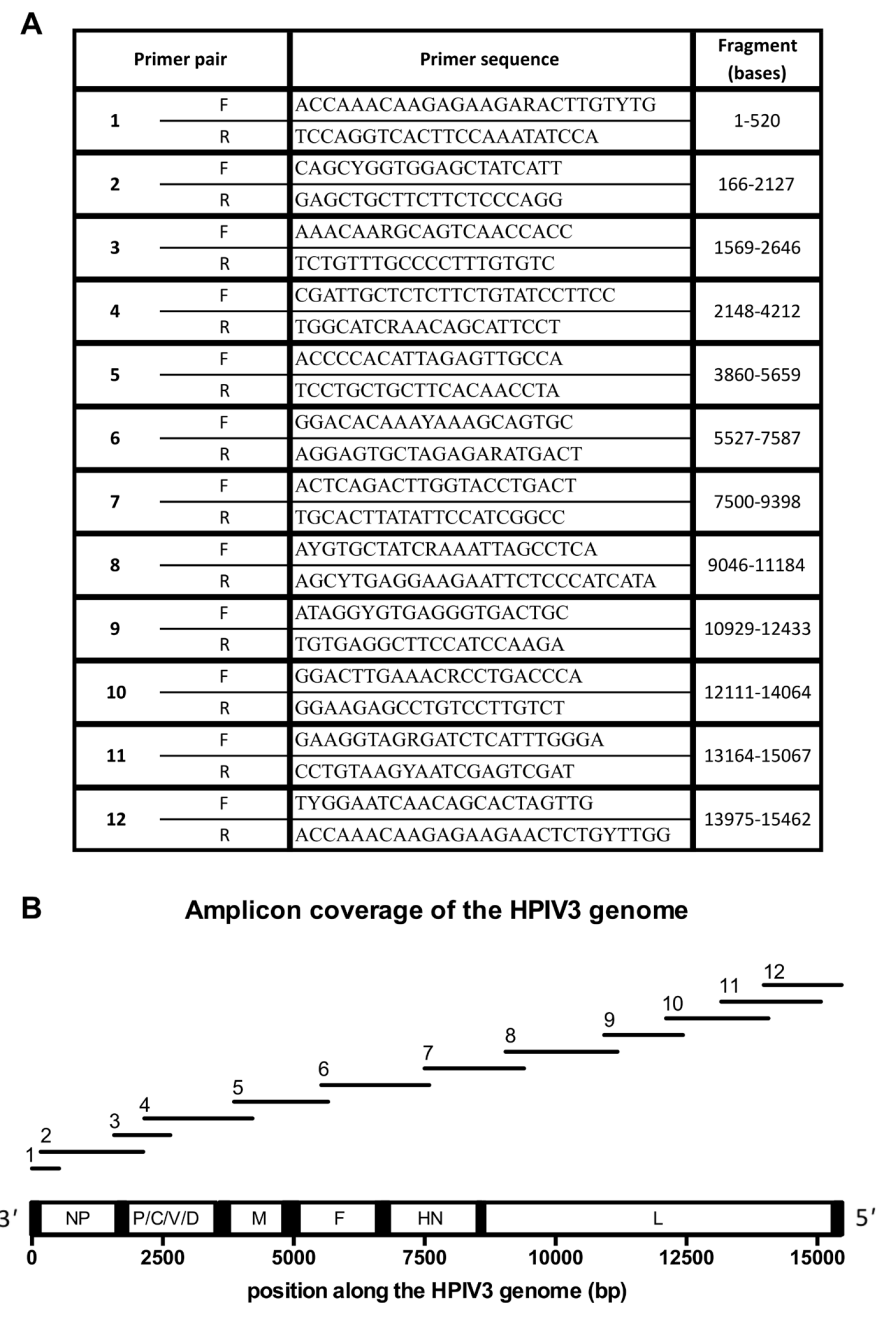
Primers used for amplicon generation for full genome sequencing ( **a**) and the position of the amplicons along the human parainfluenza viruses type 3 (HPIV3) genome. 12 primer sets ( **a**) were designed and used to generate overlapping amplicons covering the entire HPIV3 genome, as shown in ( **b**).

Total RNA from samples was extracted using the GenElute Mammalian Total RNA Miniprep kit (RTN350, Sigma) according to the manufacturer’s guidelines. Full genome amplification was achieved using a set of twelve primers producing twelve overlapping amplicons ( Figure 1).

The Superscript III One-step RT-PCR System with Platinum Taq High Fidelity from Invitrogen (12574035 Invitrogen) was used for amplicon generation. The RT-PCR was carried out on the Eppendorf Mastercycler nexus GSX1. The RT step was performed at 50°C for 30 min. This was followed by a 2min denaturation step at 94°C, and 35 cycles of denaturation (94°C for 15s), annealing (55°C for 30s) and extension (68°C 3min 30s). After the final extension step (68°C for 5min) the reaction was held at 4°C. Following amplification the products were ran on a 1% agarose gel for confirmation and purified following the Epoch Life Science Quick Protocol for EcoSpin All-in-one Mini Spin Columns (1920-050/250 Epoch Life Science).

### Reference sequence and validation of the pipeline

Two of the isolates, MK9 and 153, were first sequenced by Sanger sequencing to validate the NGS sequencing pipeline. RNA was extracted and amplicons were generated as described above. Primers were originally designed using the genscript sequencing primer design tool. These were used for Sanger sequencing (Applied Biosystems 3730xl DNA Analyser (Department of Biochemistry, University of Cambridge)) together with the amplification primers (See Dataset 1 ^20^ for primer sequences, manufactured by Eurofins) aiming for overlapping amplicons of approximately 700bp each. The sequence was then aligned to a consensus sequence of the following accession numbers KF687321, KF530255, EU326526, KF530227, KF687319, KF530232, KF687317, KF530249, KF530245, KF530250, KF530229, KF530243, AB736166, KF530252, KF530236, KF530225, KF687340, KF530254, KF687318, KF530230, KF687346, KF530233, KF530242, KF530251, KF530241, KF530253, KF530257, KF530238, KF530231, KF530234, KF530247, EU424062, KF530256, FJ455842, KF687336, U51116, NC_001796.2 (see Data availability section) using Sequencher 5.4.

### NGS sequencing and analysis

The amplicons generated were combined in equimolar concentrations and sequenced on the Illumina platform (MiSeq (Clinical Translational Research Unit, Cambridge University Hospitals, NHS Foundation Trust)). Paired short reads were then processed with Trimgalore v 0.4.2 to remove the Illumina paired end library adapters as well as short and low quality reads to retain those with length >20bp and Phred scores >20. Terminal primer sequences were subsequently removed with Cutadapt v 1.14 ^21^. Alignment was performed with Bowtie2 v 2.2.9 ^22^ using the Sanger sequences obtained above as reference and consensus was extracted with Samtools v 1.3.1 ^23^. The results were validated using Sanger sequencing of the laboratory strain MK9 and sequence 153 as well as the previously published *de novo* Ebola pipeline using QuasR v 1.20 for quality control and Spades 3.5 for assembly ^24^. Variant analysis was conducted using V-Phaser2 ^25^.

### Phylogenetics

All HPIV3 full genome sequences available on NCBI were downloaded and genomes originating from the same source and found to contain minimal variance were removed for clarity and to minimize bias. Sequences that originated from strains that were repeatedly passaged in culture or were deliberately modified, such as strain 47885, C243, 14702 and strain JS, were also removed, leaving 36 diverse full genome sequences. These, together with the 20 sequences obtained in this study were aligned in UGENE v 1.26.0 using the Muscle algorithm ^26^. Subalignments were extracted using UGENE v 1.26.0. The most suitable substitution model was selected using the JModelTest 2.0 Software ^27^. Position by position rates, distance matrices and maximum likelihood trees were generated using Molecular Evolutions Genetics Analysis (MEGA) software MEGA v 7 ^28^. Bootstrap iterations of 1000 were used for maximum likelihood tree confidence estimates. Clusters were visualized using ABGD ^15^. Bayesian Markov Chain Monte Carlo (MCMC) inference model was selected using marginal likelihood estimation using path sampling (PS) and stepping stone sampling (SS) with BEAST v 1.8.4 with a chain length of one million and one hundred path steps respectively ^29– 32^. Tracer was used to assess convergence based on the effective sample size with 10% burn-in and effective sample size (ESS) values above 200. Maximum clade credibility trees were generated with Tree Annotator and subsequently visualized and edited using FigTree v 1.4.3.

## Results

### Clinical samples and epidemiology of HPIV3

**Figure 2.**
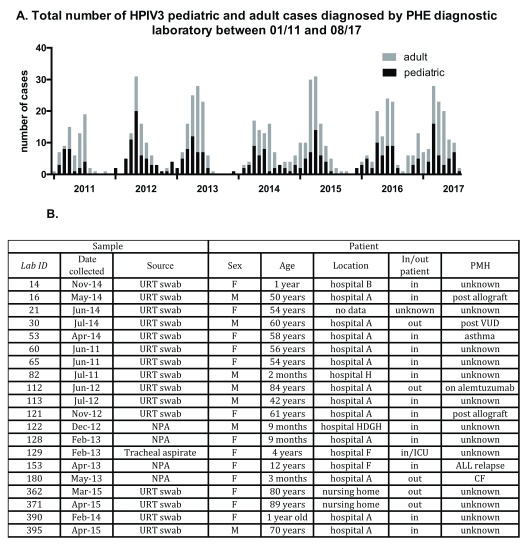
Total samples tested positive for human parainfluenza viruses type 3 (HPIV3) by Public Health England (PHE) diagnostic laboratory during 2011–2017 ( **a**) and provenance of sequenced clinical strains ( **b**). Total number of samples that have tested positive for HPIV3 in PHE diagnostic laboratory of a major teaching hospital each month are shown in ( **a**) for the period 2011–2017. The data has been extracted from the local hospital database and is separated by age. The provenance of sequenced clinical strains collected between 2011 and 2015 is shown in ( **b**). All samples originated from the upper airway and 12/20 samples were from hospital A. PMH = past medical history; NPA = Nasopharyngeal aspirate; URT = upper respiratory tract; VUD = volunteer unrelated donor (transplant); ALL = acute lymphocytic leukaemia.

The number of samples that tested positive for HPIV3 on the respiratory virus panel in PHE laboratory of a major teaching hospital during the years 2011–2017 is shown in Figure 2a.

The prevalence of HPIV3 follows a cyclical pattern with peaks occurring towards the end of spring and start of summer every year. Patient demographics for each strain are summarized in Figure 2b. The patients for which strains were sequenced represented a diverse demographic with an age distribution reflecting the usual susceptibility to HPIV3 with 18/20 being below the age of five or over the age of 50. All samples were obtained from the upper respiratory tract including swabs, nasopharyngeal aspirates and tracheal aspirates. The majority of the samples (14/20) were taken from inpatients, reflecting an unavoidable sampling bias towards cases requiring admission and potential co-morbidities. Although the majority of the cases originated from one hospital (A) (12/20), the rest were from a more diverse geographical distribution reflecting the area covered by the PHE laboratory. Relevant past medical history (PMH) is shown where available (7/20) and in most cases includes patients with haematological oncology conditions such as relapsed acute lymphocytic leukaemia (ALL), immunosuppressive chemotherapy treatment (alemtuzumab) and post bone marrow transplant including allograft and volunteer unrelated donor (VUD). This reflects the immunosuppressed population where HPIV3 is known to have the highest impact ^5, 33^. Two chronic respiratory conditions have also been identified: cystic fibrosis (CF) and asthma. Parainfluenza viruses have known to contribute to infective exacerbations of asthma ^34^, particularly in paediatrics and the clinical impact of respiratory viruses on cystic fibrosis patients is well recognised ^35^.

### Genome coverage and variant analysis

**Figure 3.**
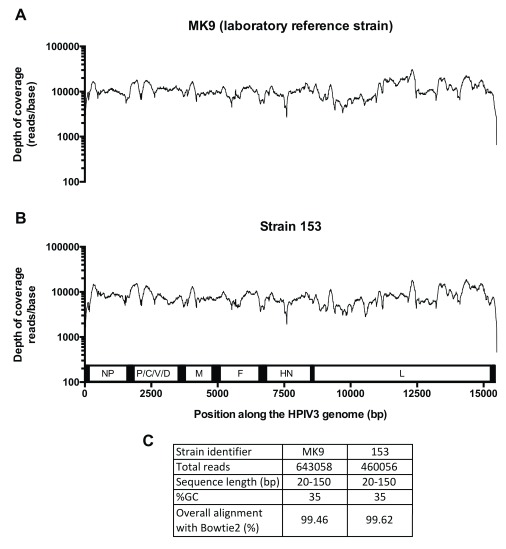
Depth of coverage achieved for laboratory strain ( **A**), strain 153 ( **B**) and FastQC ^36^ statistics for both sequences ( **C**). Consistent coverage of above 1000 was achieved over the full length of the genome of both sequences excluding the very 5′ and 3′ prime ends. The length of the final sequence was 15409 base pairs, as the forward primer (26 bases) of the first amplicon, and the reverse primer of the last amplicon (27 bases) were removed in the pipeline.

In order to evaluate the genetic variability of UK circulating strains of HPIV3, the twenty clinical strains detailed in Figure 2a and the laboratory reference strain were sequenced by NGS on the Illumina platform. The laboratory strain and strain 153 were first sequenced by Sanger sequencing and used as reference strains for NGS pipeline validation. The depth of NGS coverage for both strains as well as the average depth achieved for all the strains sequenced is shown in Figure 3.

The depth of coverage remained consistently high apart from the 5' and 3' prime ends, confirming the robustness of the pipeline. Variant analysis was then performed using V-Phaser2 and the summary results are shown in Table 1.

**Table 1.** Total number of unique minor variants detected by V Phaser2 analysis. The total number of unique minor variants detected by V Phaser 2 at each percentage level of total reads is shown. In brief, the variants are calculated both by recording the probability that a non-consensus base occurs with a greater frequency than expected by sequencing error probabilities and by analyzing he probability for non-consensus pairs of bases to co-occur given sequencing errors expected. Systematic artifacts inherent in some sequencing technologies are removed by calculating strand bias for each variant. This data is then FDR corrected and all variants with a significant (p>0.05) strand bias were excluded.

	number of variants			
*Lab ID*	<1%	1%–5%	5%–20%	>20%
14	7	0	2	0
16	4	1	0	0
21	7	3	1	1
30	3	1	1	0
53	12	7	0	0
60	9	5	1	1
65	9	5	1	0
82	2	8	4	0
112	0	6	13	0
113	4	13	22	8
121	0	3	2	0
122	9	1	0	0
128	1	1	4	1
129	1	1	0	0
153	4	3	0	0
180	4	3	23	5
362	7	0	1	0
371	4	9	1	0
390	2	1	0	0
395	5	3	1	1
MK9	36	8	1	0

### Phylogenetic analysis of the full genome sequence

**Figure 4.**
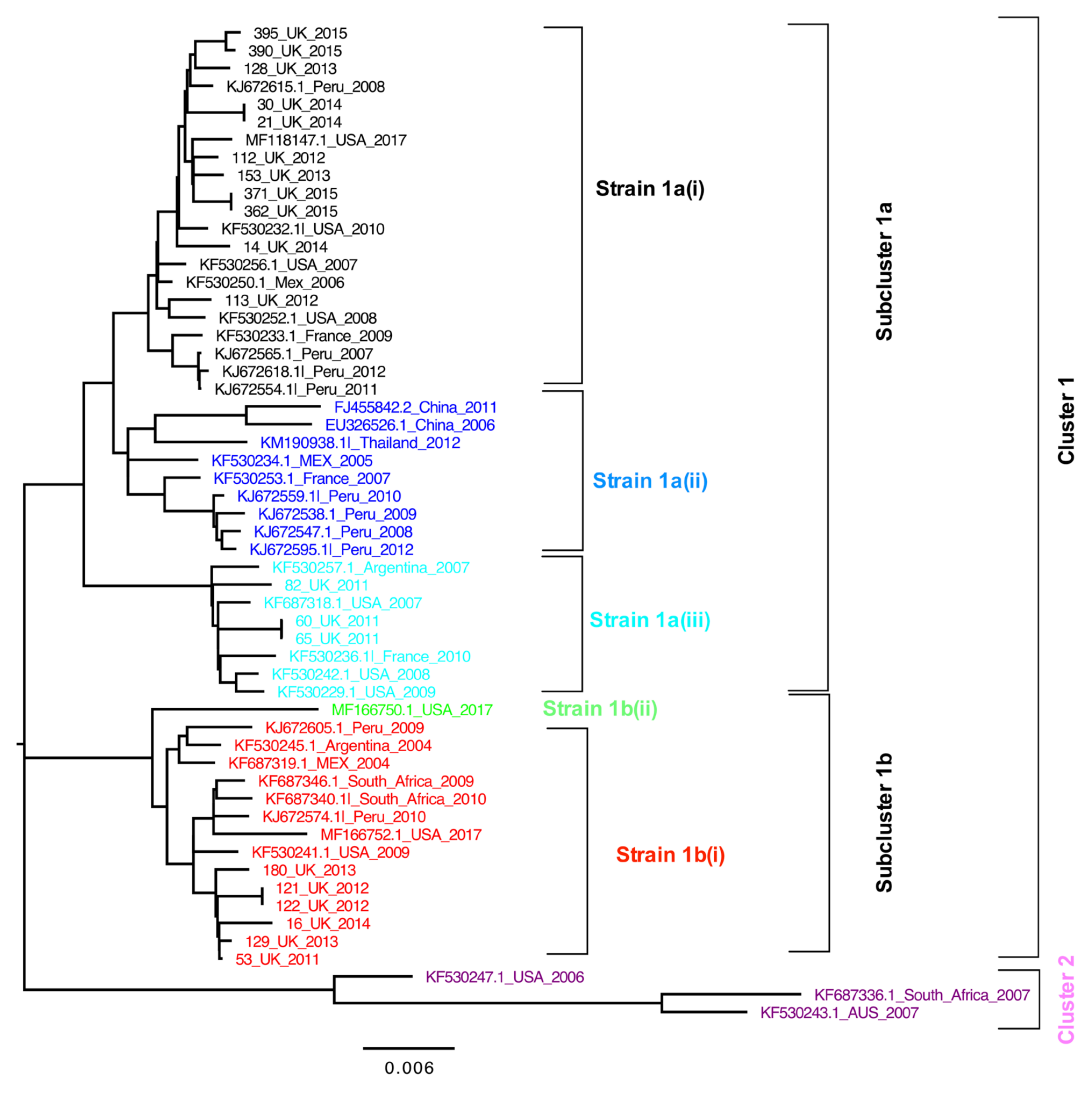
Molecular Phylogenetic analysis of human parainfluenza viruses type 3 (HPIV3) full length genome by Maximum Likelihood method. The evolutionary history was inferred by using the Maximum Likelihood method based on the General Time Reversible + I + G model and 1000 bootstrap repetitions. The tree with the highest log likelihood (-42087.04) is shown. The tree is drawn to scale, with branch lengths measured in the number of substitutions per site. The analysis involved 56 nucleotide sequences. Evolutionary analyses were conducted in MEGA7. Clusters, subclusters and strains were identified using Automatic Barcode Gap Discovery and genetic distances of 0.043 (cluster); 0.02 (subcluster) and 0.015 (strain) were identified. All strains (and cluster 2) are colored for ease of visualization and tracking.

In order to assess the epidemiology and evolution of HPIV3, in the context of strains circulating within the UK, a phylogenetic tree of full length genome sequences available was constructed using the Maximum Likelihood method ( Figure 4).

The automated barcode gap discovery analysis of the full genome sequences allowed us to define anything separated by more than a genetic distance of 0.043 as a cluster and 0.02 as a sub cluster. Therefore 2 clusters have been identified. Cluster 1 was further subdivided into subclusters, 1a and 1b, with smaller subdivisions into strains, as shown in Figure 4. It is of note that apart from strain 1b(ii) that currently only contains one full genome sequence from the USA (2017) we have not observed a temporal or geographical correlation between strains. The rate of substitution/site/year has been calculated to be 4.2 x10 ^-4^ subs/site/year using an uncorrelated relaxed clock ^37^, general time reversible model (GTR) with gamma distributed rate and invariant sites ^38– 40^ and an MCMC length of 600 million, using BEASTv1.8.4 as described in the methods. This is consistent with rates observed for other RNA viruses ^41– 44^ The average variability across the strains available was calculated using MEGA7 and found to be 2%.

### Analysis of variability along the genome can be used to identify a hypervariable region in HPIV3

**Figure 5.**
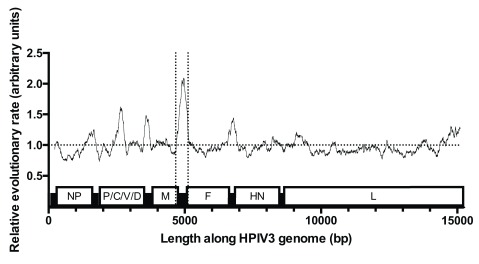
Relative site by site evolutionary rate of the human parainfluenza viruses type 3 (HPIV3) genome. Mean (relative) evolutionary rate are shown for each site next to the site number with a window of 200. These rates are scaled such that the average evolutonary rate across all sites is 1. This means that sites showing a rate < 1 are evolving slower than average, and those with a rate > 1 are evolving faster than average. These relative rate were estimated under the General Time Reversible model (+G+I). The analysis involved 56 nucleotide sequences. The position along the HPIV3 genome is shown on the x axis with the hypervariable region identified between positions 4703 to 5160. Evolutionary analyses were conducted in MEGA7.

Full genome sequencing, particularly in the context of diagnositic laboratories can be expensive and time consuming. To this end a smaller region for epidemiological and phylogentics analysis was identified by calculating relative variability rates along the HPIV3 genome ( Figure 5).

The site by site variability was calculated using the Tamura-Nei (TRN) model ^45^ of substitution with 1000 bootstrap repetitions. We have observed a peak in variability in the non-coding region between the M gene and the F gene. For the purpose of this study this region has been defined as a region of 357 base pairs in length from position 4703 to 5160 as shown in Figure 5.

### Analysis of the hypervariable region reflects the phylogenetic profile of HPIV3

**Figure 6.**
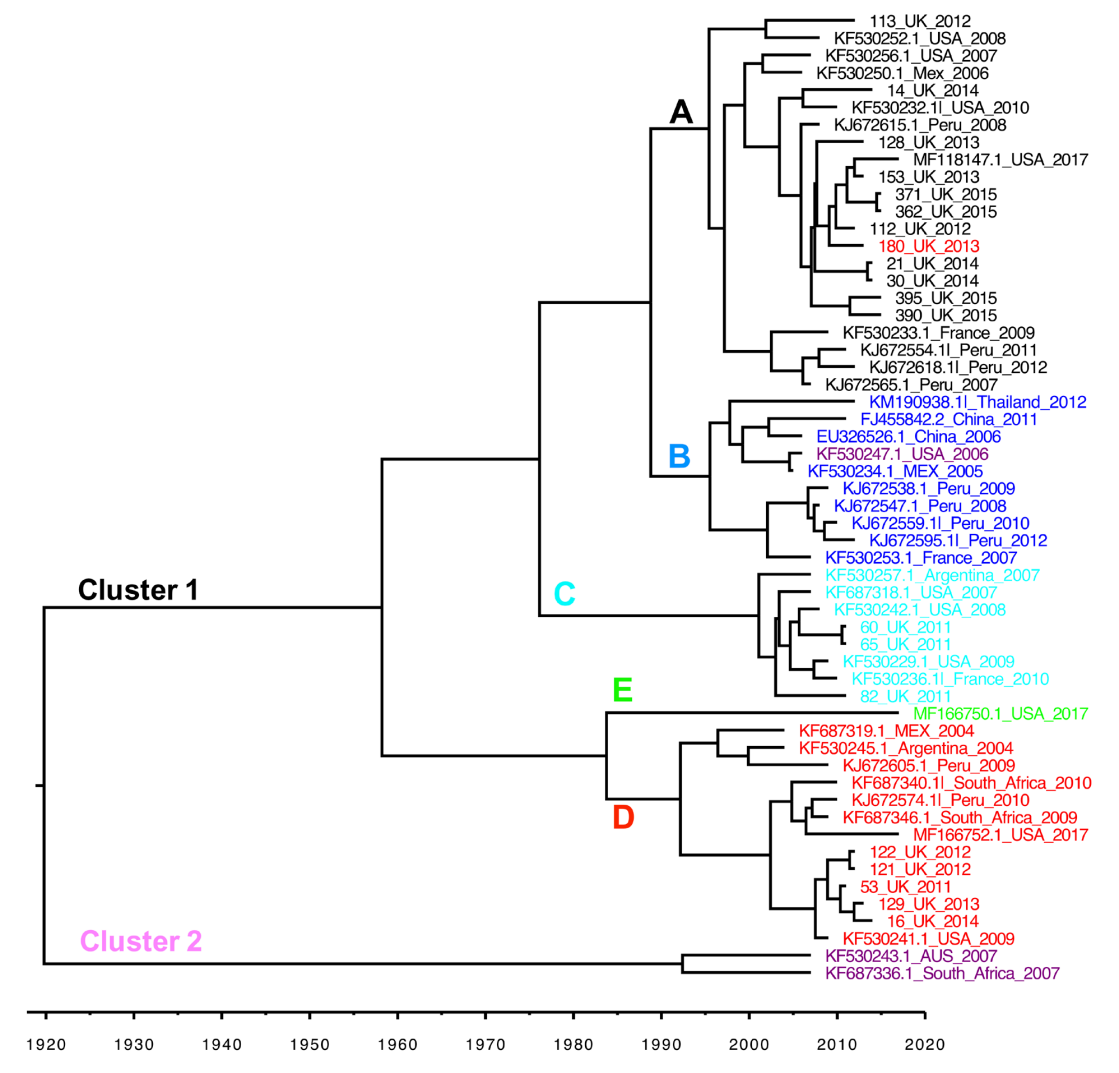
Molecular Phylogenetic analysis of human parainfluenza viruses type 3 (HPIV3) hypervariable region by Bayesian Phylogenetics using BEAST. The evolutionary history was inferred by using Bayesian Phylogenetics based on the TRN +I model using BEAST v1.8.4. The tree with the highest log likelihood (-2020.47) using path sampling and stepping stone analysis is shown. A strict clock and a constant coalescent prior were used. The MCMC length was 10,000,000. Convergence was assessed with Tracer and the maximum clade credibility tree was generated with Tree Annotator. Dates of strain emergence (in years) are shown in the figure legend. Automatic Barcode Gap Discovery was used to analyse genetic distances and 0.1 (cluster) and 0.04 (subcluster) were defined. Subclusters A-E are shown next to their respective branches. All strains and cluster and subcluster labels are colored identically to the phylogenetic analysis using full genome ( Figure 4) to demonstrate near identical clustering patterns.

The suitability of the hypervariable region for phylogentic analysis was then evaluated by constructing a phylogentic tree and comparing it to the one obtained by using full genome sequences. The BEAST evolutionary tree for the hypervariable region can be seen in Figure 6.

The rate of substitution for this region was calculated to be 1x10 ^-3^ subs/site/year with an average variability of 5%. This is markedly above the values calculated for the full genome sequence. Hence ABGD analysis ^15^ has been used to separate the sequences into subclusters corresponding to strains in the full genome analysis with a potential for finer classification for the purposes of epidemiology. The corresponding classifications are summarized in Table 2.

**Table 2.** Subdivisions identified by Automatic barcode gap discovery (ABGD) analysis of whole genome sequences of HPIV3 and hypervariable region of human parainfluenza viruses type 3 (HPIV3). The corresponding subdivisions into clusters, subclusters and strains, identified by ABGD for whole genome sequences ( Figure 4 and the hypervariable region of HPIV3 ( Figure 6) are summarized.

Subdivisions identified by ABGD analysis of				
Whole genome			Hypervariable region	
Cluster	Subcluster	Strain	Cluster	Subcluster
1	1a	1a(i)	1	A
		1a(ii)		B
		1a(iii)		C
	1b	1b(i)		D
		1b(ii)		E
2			2	

It is of note that only two strains ( Figure 4) were not classified in the same manner by both the full genome and hypervariable analysis methods. Strain 180 has moved from subcluster 1b to subcluster 1a and strain KF530247.1 (USA2006) was noted to have moved from cluster 2 to cluster 1. It was found that strain 180 contained 3.7% ambiguous bases within its hypervariable region. This falls just below the definition of a subcluster (genetic distance of 0.04) and would potentially place this sample into two subclusters depending on the alignment. This could suggest a potential co-infection with a second strain, a feature of this sample that would not have been noted if this additional analysis were not performed. Strain KF530247.1, on the other hand contains no ambiguities and therefore the migration between clusters cannot be explained this way. It has, however been identified as a potentially recombinant sequence in a previous study ^11^.

Additionally, we observed that strain 1b(ii) (MF166750.1 (USA2017)) that has been identified as an emergent strain by full genome analysis ( Figure 4), can now be defined as a subcluster in its own right, supporting the hypothesis that this may form a new emergent strain and subsequently subcluster of HPIV3 ( Figure 6).

### Analysis of the HN coding region does not fully reflect the phylogenetic profile of HPIV3 when compared to whole genome data

**Figure 7.**
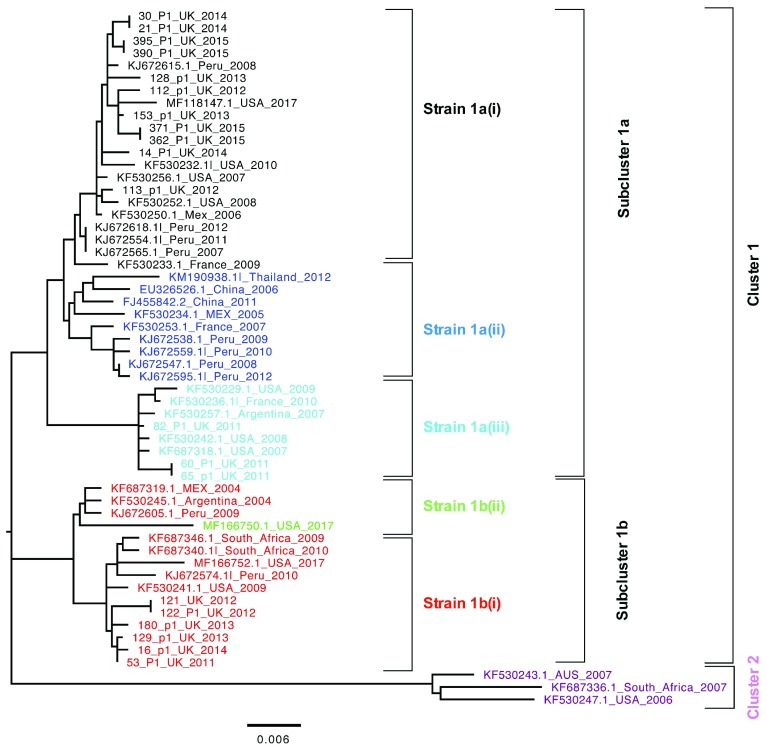
Molecular Phylogenetic analysis of human parainfluenza viruses type 3 (HPIV3) HN coding region by Maximum Likelihood method. The evolutionary history was inferred by using the Maximum Likelihood method based on the TRN +G model and 1000 bootstrap repetitions. The tree with the highest log likelihood (-4490.43) is shown. The tree is drawn to scale, with branch lengths measured in the number of substitutions per site. The analysis involved 56 nucleotide sequences. Evolutionary analyses were conducted in MEGA7. Clusters, subclusters and strains were identified using Automatic Barcode Gap Discovery and genetic distances of 0.043 (cluster); 0.02 (subcluster) and 0.01 (strain) were identified. All strains (and cluster 2) are colored consistent with Figure 4 for ease of visualization and tracking.

In order to evaluate the current findings in the context of previously published phylogenetic classification of HPIV3 ^46– 49^ an additional analysis using only the HN coding region of the HPIV3 genome was carried out ( Figure 7) and the results were compared to those obtained using whole genome sequences. Phylogenetic analysis using the F coding region was also attempted but the tree constructed did not yield clustering with high enough bootstrap values and therefore was deemed to be inadequate for the current data set. It is of note that the clustering patterns observed in Figure 7 were not wholly consistent with those obtained with whole genome data ( Figure 5) or the hypervariable region ( Figure 6). Strain 1b(ii) was found to contain 4 sequences: KF687319.1; KF530245.1; KJ67605 and MF166750.1. When using whole genome data, only the latter was classified within this strain, whereas the other sequences were clustered within strain 1b (i).

## Discussion

In this study we have presented a robust amplicon based sequencing pipeline for the evaluation of genetic variability of circulation UK strains between the years 2011–2015. The consensus sequences generated by the pipeline were validated using both Sanger sequencing and a published *de novo* pipeline ^24^. The depth of coverage obtained across all genome sequenced was consistently high ( Figure 3). Variant calling analysis was performed using VPhaser2 and the numbers of unique variants determined did not demonstrate a high degree of within host variability. This may be a reflection of the amplicon based amplification method used for this pipeline as capture-based methods have been shown to provide a more robust minor variant amplification approach ^50, 51^. However amplicon based methods have previously been used successfully for variant calling in a clinical setting ^52^ and have been considered to be effective for minority variable identification. On the other hand, as all the sequenced strains were grown from clinical samples in immortalized cell tissue culture, it most likely reflects the genetic bottleneck due to this method ^53^.

The clinical strains sequenced were drawn from a wide demographic with an inevitable bias towards inpatient admissions due to increased viral testing in these circumstances. The sample demographic reflected known populations with a known severe impact by HPIV3. 18 out of 20 strains were obtained from patients aged below 5 or over 50. Although clinical data was only available in seven cases, two patients were identified as having chronic respiratory conditions and five were immunosuppressed due to a haemato-oncological condition. Although this sample formed a good representation of the population most commonly severely affected by HPIV3 ^33– 35^, further studies focusing on the occurrence of HPIV3 in the otherwise healthy cohort could enhance the knowledge of its variability.

To this end the phylogenetic analysis of HPIV3 UK sequences obtained was conducted in the context of other full genome sequences currently available in the NCBI database. We have excluded all duplicate and highly similar sequences as well as significantly culture adapted strains. Consequently we have observed only two clusters, which contrasts with three previously identified in literature ^54^. This is most likely due to the exclusion of strains from the last century that are known to be heavily culture adapted and have therefore not been included in this study. We have observed that cluster 1 ( Figure 4) contains most strains that are currently circulating including all the UK strains sequenced in this study. Cluster 1 can further be subdivided into two subclusters with UK strains falling into both rather than forming a UK specific subcluster or strain.

We have not noted any significant geographical or temporal correlation between strains and clusters using the limited number of diverse sequences available to us ( Figure 4). The majority of the strains were observed at different geographical locations and with a wide temporal spread. This reflects that HPIV3 is a global problem that has been identified as an important respiratory pathogen in all countries conducting respiratory virus surveillance ^4^. The rate of substitution for HPIV3 (4.2x10 ^-4^ subs/site/year) is consistent with other RNA viruses ^41, 42^ and would predict a development of a new subcluster roughly every 50 years and a new cluster every 107 years. We have observed a potential new emerging strain MF166750.1 (USA2017), and a strain (1a(ii)) that has not appeared since 2012 ( Figure 4). Although the rate of evolution, in itself, would be insufficient to account for the yearly rise in cases seen, it does reflect a number of globally circulating strains as well as regularly emerging new ones.

Limited geographical clustering observed in this analysis was most likely due to the scarcity of diverse sequences from specific geographical regions, particularly for South/East Asia. Over the last few years, this number has increased, partially due to decreased cost and greater access to next generation sequencing ^55^ and a greater interest in viral metagenomics of respiratory infections ^56^. However the bulk of whole genome sequences available centers on a small number of episodes or studies, yielding clusters of highly similar or identical genomes ^57^. Most historical sequences used for previous phylogenetic analyses ^46– 48^, omitted in this study, have been propagated and maintained in cell culture prior to sequencing ^58– 60^ Cell culture adaptation of HPIV3 has been well studied both phenotypically, where culture adapted strains were shown to produce larger plaques in cell culture as well as genotypically, with a particular focus on HN and F coding regions, where it has been shown that different adaptations are required for growth in cell culture as opposed to the natural host ^61– 63^.

It could be argued that some degree of adaptation was also inherent in the strains used in this study, as they have undergone one passage in PLC/PRF/5 cells prior to sequencing. However the sequences obtained were minimally adapted compared to historical strains maintained in cell culture over many passages ^64^. Additionally these clinical sequences were found to be phylogenetically diverse and to cluster with other clinical strains of HPIV3, whereas significantly culture adapted strains have previously been shown to form a separate cluster, as described above. An alternative approach to whole genome sequencing, using the small hypervariable region, easily sequenced from clinical samples was also explored in this paper, and could be used for phylogenetic typing of HPIV3 where whole genome data cannot be obtained.

We have demonstrated that a salient phylogenetic and epidemiological analysis can be performed on a short hypervariable region of the HPIV3 genome described in this study. Although an overlapping region has been used previously for outbreak monitoring, it was a shorter segment of 244 bases, position 4880 to 5124 ^8, 9^. This would place the primer sites within the hypervariable region itself (defined here as position 4703 to 5160, Figure 3), potentially limiting amplification success rate. In the current analysis the region identified is flanked by two regions of reduced variability, creating ideal locations for primer localization ( Figure 5).

A faster substitution rate of the hypervariable region (1x10 ^-3^) has facilitated the division into 2 clusters and 5 further subclusters. All strains apart from two, have been shown to cluster in an identical pattern to that identified in the full genome analysis. MF166750.1 (USA2017) has been identified as forming its own subcluster, which was consistent with full genome analysis, where it was identified as an emerging strain ( Figure 2). This is particularly salient in a clinical environment where whole genome sequencing may not be feasible due to the quality and storage of the clinical samples. The costs and turn around times associated with next generation sequencing are often prohibitive in a diagnostic laboratory. The comparatively short hypervariable fragment can be sequenced by Sanger sequencing, decreasing costs, turn around times and removing the need for complex bioinformatics analysis as demonstrated in the companion paper ^65^.

It is of note that only three full genome strains were found to fall into cluster 2 when full genome phylogenetic analysis was conducted and one of the strains (KF530247.1) moved to cluster 1 when only the hypervariable region was used for analysis ( Figure 2 and Figure 4). KF530247.1 was previously identified in literature as a potential recombinant strain, where phylogenetic analysis using its HN genome did not correlate with that of the F genome ^11^. It is important to note that this strain and the two remaining strains form part of the same sequencing project, accession number PRJNA73055, however other strains from the same project behaved as predicted in our analysis. Hence, this likely reflects the scarcity of full genome data available for the analysis of HPIV3. Overall, the geographical distribution of sequences available was noted to be heavily biased towards the northern hemisphere and the Americas. This highlights the necessity to conduct further epidemiological surveillance of HPIV3 as well as the utility of a smaller part of the genome that could be used for this purpose, as identified in this study.

The second sample that did not cluster in the same fashion as that predicted by full genome analysis was UK strain 180. Further analysis of the sequence revealed that there were 3.7% ambiguous bases in the hypervariable region of this strain. This ambiguity could influence the subcluster (genetic distance 0.043) in which this strain would have been grouped. As a number of different strains have been shown to circulate globally each year, we could hypothesise that this is a case of dual infection that was identified when the analyses of the hypervariable region and the full genome were compared.

As the HN coding region has previously been used for phylogenetic characterization of HPIV3 ^46– 49^ this approach was also applied to the current data set ( Figure 7). It is interesting to note that the resulting clustering patterns were not wholly consistent with those obtained by whole genome sequencing. Three strains (KF687319.1; KF530245.1; KJ67605) were classified differently ( Figure 7). However both strains 180 and strain KF530271.1 were classified in agreement with whole genome analysis ( Figure 5). The discrepant strains in this case were sourced from different projects, have not been previously identified in literature as atypically clustering, and did not contain sufficient ambiguous bases that could have potentially influenced the classification, as described above for strains 180 and KF530271.1 Unfortunately, without further understanding of the connection between genotype and clinical and evolutionary phenotype of HPIV3, it is impossible to infer which analysis produced more biologically salient results. However we have shown that phylogenetic analysis using HN did not fully reflect the clustering obtained using whole genome data for this data set and this was matched more closely by the hypervariable region identified in this study. 

In conclusion, in this study we have presented a first genetic analysis of whole genome sequences of circulating UK strains of HPIV3 in the period of 2011–2015. We have concluded that, consistent with previous data, the circulating strains fall into the same cluster but do not form a particular subcluster or strain ^11– 13, 54^. Within the constraints of the amount of sequences available we have not observed a temporal or geographical correlation with a particular strain of HPIV3. However the number of full genome HPIV3 sequences is limited, necessitating further epidemiological investigation both in a laboratory and clinical setting. To this end, we have identified a small hypervariable region in the HPIV3 genome and have shown that, in the absence of full genome data, this region can be used for epidemiological surveillance of HPIV3.

## Data availability

Raw data and details of primers used for Sanger sequencing available on Open Science Framework. Dataset 1: UK circulating strains of human parainfluenza 3: an amplicon based next generation sequencing method and phylogenetic analysis. https://doi.org/10.17605/OSF.IO/SUQ95 ^20^

All HPIV3 sequences have been uploaded to NCBI with the following accession numbers:

HPIV3/UK/ **65**/06/2011 MH678674

HPIV3/UK/ **112**/06/2012 MH678675

HPIV3/UK/ **113**/07/2012 MH678676

HPIV3/UK/ **121**/11/2012 MH678677

HPIV3/UK/ **128**/02/2013 MH678678

HPIV3/UK/ **129**/02/2013 MH678679

HPIV3/UK/ **153**/04/2013 MH678680

HPIV3/UK/ **180**/05/2013 MH678681

HPIV3/UK/ref/ **MK9** MH678682

HPIV3/UK/ **14**/11/2014 MH678683

HPIV3/UK/ **16**/05/2014 MH678684

HPIV3/UK/ **21**/06/2014 MH678685

HPIV3/UK/ **30**/07/2014 MH678686

HPIV3/UK/ **60**/06/2011 MH678687

HPIV3/UK/ **82**/07/2011 MH678688

HPIV3/UK/ **122**/12/2012 MH678689

HPIV3/UK/ **362**/03/2015 MH678690

HPIV3/UK/ **371**/04/2015 MH678691

HPIV3/UK/ **390**/02/2014 MH678692

HPIV3/UK/ **395**/04/2015 MH678693

HPIV3/UK/ **53**/04/2014 MH678694

All uploaded sequences can be accessed together via PopSet: 1470015825
