# Supplementary material for: UK circulating strains of human parainfluenza 3: an amplicon based next generation sequencing method and phylogenetic analysis
Source: Wellcome Open Res. 2018 Nov 26;3:118. Originally published 2018 Sep 19. [Version 2] doi: 10.12688/wellcomeopenres.14730.2 (PMC6281019; doi:10.12688/wellcomeopenres.14730.2)
Supplement: Peer review history

# Peer review: Referee response for version 2

**DOI:** 10.21956/wellcomeopenres.16293.r34334

**Published:** 2018-12-03

**Authors:** Hirokazu Kimura

**Affiliations:** 1 School of Medical Technology, Faculty of Health Sciences, Gunma Paz University, Gunma, Japan; 2 Graduate School of Health Science, Gunma Paz University, Gunma, Japan

The  authors addressed all my comments. Thus, the revised manuscript is acceptable for indexing.

I have read this submission. I believe that I have an appropriate level of expertise to confirm that it is of an acceptable scientific standard.

# Peer review: Referee response for version 2

**DOI:** 10.21956/wellcomeopenres.16293.r34333

**Published:** 2018-11-28

**Authors:** Julian Wei-Tze Tang

**Affiliations:** 1 Clinical Microbiology, University Hospitals of Leicester NHS Trust, Leicester, UK; 2 University of Leicester, Leicester, UK

I have read this submission. I believe that I have an appropriate level of expertise to confirm that it is of an acceptable scientific standard.

# Peer review: Referee response for version 1

**DOI:** 10.21956/wellcomeopenres.16048.r33935

**Published:** 2018-10-10

**Authors:** Julian Wei-Tze Tang

**Affiliations:** 1 Clinical Microbiology, University Hospitals of Leicester NHS Trust, Leicester, UK; 2 University of Leicester, Leicester, UK

A very detailed, comprehensive write-up from the NGS methods viewpoint – which seems to be

 the main focus of this paper/studentship.

 But a more global contextualization would make the paper more interesting and relevant to an

 international readership – this is very brief at present:

 “Overall, the geographical distribution of sequences available was noted to be heavily biased

 towards the northern hemisphere and the Americas. This highlights the necessity to conduct

 further epidemiological surveillance of HPIV3 as well as the utility of a smaller part of the genome

 that could be used for this purpose, as identified in this study.”

 Especially in the Discussion where the UK sequences cluster with the other North/South American

 and European sequences. Why is there no clustering with South/East Asian PIV3 sequences? Were

 they too few or just not included in the alignment – or some other reason? See:

 Godoy *et al.* (2016) ^1^ 

 Kim *et al*. (2017) ^2^

 Almajhdi *et al.* (2012) ^3^

 Piralla *et al.* (2009) ^4^

 Zhang *et al.* (2013) ^5^

 The authors should also discuss the possible impact of cell-cultured PIV3 viruses and how this may

 impact on the sequence fidelity in the resulting sequence obtained. This has been a problem for

 influenza viruses cultured in MDCK cell-lines for sequencing – and probably applies to other

 respiratory viruses cultured/amplified for sequencing:

 Lee *et al.* (2013) ^6^

 Also as well as just analyzing the whole genome sequence phylogenetically, the authors should

 also analyse the individual PIV gene sequences ( at least where sufficient numbers of comparative

 sequences are available on Genbank) to see if the same cluster pattern is maintained compared to

 the whole genome phylogenetic analysis they have performed already. If the individual gene

 sequence analyses show different topologies, this may indicate some increased variability/ hypermutation/

 recombination ongoing in some genes more than others, e.g. the fusion F gene. This

 data may impact on vaccine design considerations, where a globally conserved region is an

 important target consideration - see:

 Tang *et al. *(2017) ^7^

I have read this submission. I believe that I have an appropriate level of expertise to confirm that it is of an acceptable scientific standard.

## References

[1] GodoyCPeremiquel-TrillasPAndrésCGimferrerLUrionaSMCodinaMGArmadansLMartínMdel CFuentesFEsperalbaJCampinsMPumarolaTAntónA: A molecular epidemiological study of human parainfluenza virus type 3 at a tertiary university hospital during 2013-2015 in Catalonia, Spain. *Diagn Microbiol Infect Dis*.2016;86(2) : 10.1016/j.diagmicrobio.2016.07.023 153-9 10.1016/j.diagmicrobio.2016.07.023 27524509PMC7127006

[2] KimTJinCESungHKooBParkJKimSMKimJYChongYPLeeSOChoiSHKimYSWooJHLeeJHLeeJHLeeKHShinYKimSH: Molecular epidemiology and environmental contamination during an outbreak of parainfluenza virus 3 in a haematology ward. *J Hosp Infect*.2017;97(4) : 10.1016/j.jhin.2017.09.003 403-413 10.1016/j.jhin.2017.09.003 28893615PMC7114920

[3] AlmajhdiFNAlshamanMSAmerHM: Molecular characterization and phylogenetic analysis of human parainfluenza virus type 3 isolated from Saudi Arabia. *J Med Virol*.2012;84(8) : 10.1002/jmv.23326 1304-11 10.1002/jmv.23326 22711360

[4] PirallaAPercivalleEDi Cesare-MerloneALocatelliFGernaG: Multicluster nosocomial outbreak of parainfluenza virus type 3 infection in a pediatric oncohematology unit: a phylogenetic study. *Haematologica*.2009;94(6) : 10.3324/haematol.2008.003319 833-9 10.3324/haematol.2008.003319 19377073PMC2688575

[5] ZhangPJiYXXuSTYuDSYuPBZhangHXuJCuiALWeiHLXuWB: [Genetic characterization of human parainfluenza virus 3 circulating in Gansu and Shaanxi Provinces from 2009 to 2011]. *Bing Du Xue Bao*.2013;29(5) :509-14 24386839

[6] LeeHKTangJWKongDHLohTPChiangDKLamTTKoayES: Comparison of mutation patterns in full-genome A/H3N2 influenza sequences obtained directly from clinical samples and the same samples after a single MDCK passage. *PLoS One*.2013;8(11) : 10.1371/journal.pone.0079252 e79252 10.1371/journal.pone.0079252 24223916PMC3815150

[7] TangJWLamTTZaraketHLipkinWIDrewsSJHatchetteTFHeraudJMKoopmansMPINSPIRE investigators: Global epidemiology of non-influenza RNA respiratory viruses: data gaps and a growing need for surveillance. *Lancet Infect Dis*.2017;17(10) : 10.1016/S1473-3099(17)30238-4 e320-e326 10.1016/S1473-3099(17)30238-4 28457597PMC7164797

# Response

**Published:** 2018-11-21

**Authors:** Anna Smielewska

**Affiliations:** University of Cambridge Addenbrooke's Hospital Cambridge, UK

We would like to thank Dt Tang for his time and expertise and for the helpful comments. These have been addressed in version 2 of the manuscript as follows:

1. A note on geographical clustering has been added in Discussion 

2. A note on the effects of cell culture adaptation has been added in Discussion 

3. Analysis of the HN coding region has been included – additional figure (Figure 7) added in Results and a paragraph added in Discussion

4. Analysis using the F coding region was attempted, however this did not yield clustering with high enough bootstrap values and therefore was deemed to be an inadequate method for the current data set. A note to this effect has been added in Results.

# Peer review: Referee response for version 1

**DOI:** 10.21956/wellcomeopenres.16048.r33936

**Published:** 2018-10-03

**Authors:** Hirokazu Kimura

**Affiliations:** 1 School of Medical Technology, Faculty of Health Sciences, Gunma Paz University, Gunma, Japan; 2 Graduate School of Health Science, Gunma Paz University, Gunma, Japan

The authors performed detailed genetic analyses of human respirovirus 3 (HRV3) strains detected in UK using primer walking combined with the NGS method. I feel that methodology is sound. Moreover, the present genetic and molecular epidemiological data may be partly contribute to understand HRV3 virology. However, some major concerns were found.

 1. HPIV3 has been reclassified into another Genus (Respirovirus) and species (respirovirus 3) by ICTV. It should be amended.

 2. The authors phylogenetically analyzed using full genome of the present strains. Why?

 3. No discussion of the phylogenetic relationships between your country and other countries was seen ^1^^-^^2^. The authors could refer to them.

 4. The present strains could be genotyped and/or subgenotyped as previously described ^3^.

    

I have read this submission. I believe that I have an appropriate level of expertise to confirm that it is of an acceptable scientific standard, however I have significant reservations, as outlined above.

## References

[1] MizutaKTsukagoshiHIkedaTAokiYAbikoCItagakiTNaganoMNodaMKimuraH: Molecular evolution of the haemagglutinin-neuraminidase gene in human parainfluenza virus type 3 isolates from children with acute respiratory illness in Yamagata prefecture, Japan. *J Med Microbiol*.2014;63(Pt 4) : 10.1099/jmm.0.068189-0 570-7 10.1099/jmm.0.068189-0 24464692

[2] TakahashiMNagasawaKSaitoKMaisawaSIFujitaKMurakamiKKurodaMRyoAKimuraH: Detailed genetic analyses of the HN gene in human respirovirus 3 detected in children with acute respiratory illness in the Iwate Prefecture, Japan. *Infect Genet Evol*.2018;59: 10.1016/j.meegid.2018.01.021 155-162 10.1016/j.meegid.2018.01.021 29408530

[3] AlmajhdiFN: Hemagglutinin-neuraminidase gene sequence-based reclassification of human parainfluenza virus 3 variants. *Intervirology*.2015;58(1) : 10.1159/000369208 35-40 10.1159/000369208 25592955

# Response

**Published:** 2018-11-21

**Authors:** Anna Smielewska

**Affiliations:** University of Cambridge Addenbrooke's Hospital Cambridge, UK

We would like to thank Dr Kimura  for his time and expertise and for the helpful comments. These have been addressed in version 2 of the manuscript as follows:

 1. A note on renaming of human parainfluenza virus 3 as human respirovirus 3 has been added in Introduction. 

 2. Additional clarification on why whole genome sequencing was used was added in Introduction 

 3. A note on geographical clustering has been added in Discussion 

 4. Analysis of the HN coding region was carried out, an additional figure (Figure 7) was added in Results and a paragraph added in Discussion.
